# A New Invalid Bed

**Published:** 1868

**Authors:** 


### A NEW INVALID BED.

There is now on view at the establishment of Mr. Ward, the invalid chair-maker, Leicester Square, a new invalid bed, admitting of a much greater variety of movements than any of those at present in use. The upper framework has adjustments similar to those of an ordinary fracture-bed, permitting the body to be raised to various inclinations, and the knees to be bent to various angles. But the peculiarity is, that this frame-work is supported, under its centre, on a large ball and socket-joint, which allows the whole frame-work, with its variously adjustable ports, to be moved about bodily in all directions, so as to be inclined longitudinally, laterally, or both, and to be moved round so as to face all points of the compass. By means of a simple locking apparatus, the framework is firmly fixed in any attitude that may be desired ; a few turns of the handle sufficing again to release it, and any other attitude to be assumed. Among the advantages obtained are these :

The patient may be taken out of bed and put into bed again without the effort ordinarily required. The ball being unlocked, and the bed being gently tipped forward, so that its lower end reaches the floor, the patient comes upon his feet; and after the sheets have been changed, or some needful act performed, he is placed with his back against the inclined surface of the bed, which, being then made to revolve backward, he lies as at first.

By a lateral instead of a longitudinal inclination of the bed, the patient may be turned over from the back on to the side, or contrariwise, saving the labor and pain often entailed by this change.

The longitudinal inclination of the bed being changable at pleasure, the patient may lie or may sleep at any angle that he may prefer, or that is prescribed, either with the head higher than the feet, or, as it is sometimes desirable, with the feet somewhat higher than the head; the inclination being, of course, adjustable to nicety and changable at will.

The movable framework which supports the trunk being raised, so that the trunk and legs form an angle (which may be varied to any extent up to a right angle), the whole bed may then be moved longitudinally round its centre of support, so that the body in this bent position may have the head and feet placed at all varieties of relative elevation.— For example, while the trunk is horizontal, the legs may be greatly inclined upward, an attitude that is desirable where injury of the foot or knee renders it proper to diminish the pressure of blood.

The framework that bends the knees being raised, as well as that which inclines the trunk, the same longitudinal rotation of the framework gives a great variety of partly reclining, partly sitting postures. The patient may be placed, without any effort to him, in all attitudes between that of lying horizontally and that of sitting upright in an easy chair.

These movements may, of course, be all of them joined with any such degree of lateral inclination of the bed as is desired; so that, supposing the framework has been adjusted somewhat into the form of an easy chair, and tilted forward or backward so as to bring a wounded arm or foot to the right height, the bed may be at the same time tilted sideways, so as to bring this wounded arm or foot on the uppermost side, into the most convenient position for dressing the wound.

At the same time the movement of horizontal rotation being brought into play, the whole bed may be moved round until the injured part is turned toward the light; this same horizontal rotation being, at other times, available for giving the patient change of view, enabling him to look out of the window when raised in the sitting posture, or to have his face turned away from the light if it is distressing.

To the side of the framework is fixed a movable arm, carrying a small table to support a plate or basin, and this, by a slight change of position, also becomes a reading easel.

One of the advantages of the bed, not originally foreseen, but which has come out in practice, is that of being able to make certain changes in a patient's position quite suddenly. When the ball and socket-joint is but partially locked, so that a moderate force applied to the head or foot of the bed will change its position, the patient, previously lying back, may be instantly raised into the sitting posture if a coughing fit come on.

One further use that may be named is, that when the ball and socket-joint is completely unlocked, so as to permit perfect freedom of movement, two attendants, seizing the handles on the opposite sides of the bed, may give the patient a little exercise by rocking the bed from side to side in the manner of a cradle.

Beyond the special advantages above described, there are some general advantages. The ability to change the posture of the patient in such a variety of ways and degrees, without any effort to him, must tend to diminish that pain, weariness and irritability caused by long continuance of the same attitude, or by small choice of attitudes, and must so conduce to convalescence. A further result to be anticipated is, that bed-sores may be avoided, the points of chief pressure being changeable at will, and as often as is desired.

This bed, devised by Mr. Herbert Spencer, the distinguished biologist and philosophical writer, for a member of his own family, has been in use between four and five months, and has so far answered expectations that he has had a second made, with sundry improvements, hoping that it may be of service to others. Mr. Spencer has refrained from patenting it, not wishing to place any obstacle in the way of its general use.—*British Medical Journal.*

